# Complete Genome Sequences of Cluster G Mycobacteriophage Darionha, Cluster A Mycobacteriophage Salz, and Cluster J Mycobacteriophage ThreeRngTarjay

**DOI:** 10.1128/MRA.00160-20

**Published:** 2020-05-14

**Authors:** Andrea M. Sandoval, Amber M. Abram, Zahraa M. Alhabib, Angelina S. Antonyan, Salar M. Brikho, Sarah I. Buhay, Griffin E. Craig, Karen G. Crile, Nour El Yaman, Lizbeth Garcia-Leon, Zahraa B. Hammoud, Anthony R. Huffman, Ali H. Issa, Alexander B. Jackman, Victoria K. Krajcz, Yamiya J. Lloyd, Marcel L. Jones, Diana L. McMahon, Briana A. D. Murdock, Jada J. Nelson, Tulsi T. Patel, Yashodhara V. Patil, Sabriyyah A. Ricketts, Leonardo S. Romero-Barajas, Laila H. Sareini, Channing S. Sesoko, Marcelio A. Shammami, Erin E. Sheardy, John R. Sherwood, Arren E. Simpson, Racha H. Tiba, Stephanie B. Conant, Jonathan S. Finkel, Jacob D. Kagey

**Affiliations:** a Department of Biology, University of Detroit Mercy, Detroit, Michigan, USA; b ReBUILDetroit, University of Detroit Mercy, Detroit, Michigan, USA; Queens College

## Abstract

Mycobacteriophages Darionha, Salz, and ThreeRngTarjay are mycobacteriophages isolated using the host Mycobacterium smegmatis mc^2^155. Following isolation from soil samples, all three siphoviridae phages were characterized, and their genomes were sequenced and annotated.

## ANNOUNCEMENT

Mycobacteriophages Darionha, Salz, and ThreeRngTarjay are bacteriophages isolated from soil found at the University of Detroit Mercy campus in Detroit, Michigan, using the host Mycobacterium smegmatis mc^2^155. M. smegmatis is a well-known actinobacterium that has been demonstrated to be a useful host for isolating this type of phage (1). The isolation, characterization, sequencing, and annotation of each phage was done as part of the Science Education Alliance–Phage Hunters Advancing Genomics and Evolutionary Science (SEA-PHAGES) program. The SEA-PHAGES database has over 2,900 completely sequenced phage genomes currently listed (2, 3).

The three mycobacteriophages were found using either direct plating or enriched soil samples with the host M. smegmatis, which is a host that is widely used by the SEA-PHAGES community. M. smegmatis was grown on 7H9 medium supplemented by albumin and dextrose (AD) at 37°C. ThreeRngTarjay was found from direct plating, and Salz and Darionha were found from enriched soil samples. Following isolation, DNA was isolated using the Wizard DNA cleanup kit (Promega). The genomes were sequenced at the University of Pittsburgh using the Illumina MiSeq platform v3 and assembled using Newbler v2.9 and Consed v29.0 (4, 5). Darionha had 991,310 individual reads with approximately 3,342-fold shotgun coverage. Salz had 796,129 individual reads with approximately 2,179-fold shotgun coverage. ThreeRngTarjay had 281,178 individual reads with approximately 350-fold shotgun coverage. Phage genomes were checked for completeness and assembled, and genomic termini were determined using Newbler and Consed v29 as done previously (6). The genomes were annotated using DNA Master v5.22.3 (7), Glimmer v3.02 (8), GeneMark v2.5 (7), Starterator (https://seaphagesbioinformatics.helpdocsonline.com/home), Phamerator v3 (9), HHpred v2.07 (10), and BLASTp v2.7.1 (11, 12). Default parameters were used for all software except that specific parameters for DNA Master, HHpred, and BLASTp were used as previously described, namely, an E value cutoff of 10e-4 was used for HHpred and BLASTp (3). General mycobacteriophage features of each genome are listed in Table 1.

**TABLE 1:** Features of the three mycobacteriophages, isolated in Detroit, Michigan

Phage name	GenBank accession no.	SRA accession no.	Length (bp)	G+C content (%)	No. of ORFs^*a*^	No. of tRNAs	Cluster	Life cycle
Darionha	MK524493	SRX7690243	41,901	66.57	65	0	G1	Temperate
Salz	MK524519	SRX7690248	51,333	63.85	94	1	A11	Temperate
ThreeRngTarjay	MK524527	SRX7690249	113,254	60.96	240	1	J	Temperate

a ORFs, open reading frames.

Based on the nucleotide similarity between other phages isolated from the same host, phages can be grouped based on sequence similarity into clusters for any phage sharing sequence similarity with >50% of their genome (13). Using these criteria, ThreeRngTarjay was classified as a cluster J phage. Phages in this cluster are *Siphoviridae* despite having unusually long genomes, with an average genome size of 111,009 bp. ThreeRngTarjay has a genome size of 113,254 bp, following the trend of cluster J phages. Cluster J phages are also unique in their mosaic genomic properties and architecture, gene functions, capsid structure, gene mobility, and intron splicing (11, 12).

Salz was classified as a cluster A phage, the largest cluster (14), and further categorized into subcluster A11. Cluster A phages are similar in size and genomic organization and share a homologous immunity system (15, 16). This immunity system is unique in cluster A in that these phages have superinfection immunity. Additionally, cluster A phages encode a repressor protein, which for Salz is gp72. The subcluster designation was determined on the variation of this stoperator sequence (gene 46), which categorizes Salz into subcluster A11 (15, 16).

Darionha was classified as a cluster G phage. Subcluster G1 phages are distinct and unique from other cluster G phages based on a centrally located immunity cassette (integrase and repressor) required for integration-dependent immunity (14, 17). For Darionha, the integrase and repressor are located next to each other on genes 32 and 33, respectively. These two genes are what define and give the phage its lysogenic properties and its prophage stability (14). Lysogeny was confirmed through the plaque morphology of a characteristic incomplete clearing of the bacterial host.

We deem the annotation of these genomes to be complete. Each genomic region was annotated independently by two groups of annotators, and any differences were reconciled. The annotated genomes were then sent to SEA-PHAGES for further quality control before submission to GenBank and SRA.

### Data availability.

The GenBank and SRA accession numbers are listed in Table 1.
